# SLC25A37 as a novel therapeutic target for benign prostatic hyperplasia: integrative analyses of single-cell RNA sequencing and genome-wide association studies

**DOI:** 10.1515/med-2025-1371

**Published:** 2026-01-13

**Authors:** Zhen Tong, Wei Zhang, Xinrui Wu, Xinxing Du, Zehong Peng, Liang Dong, Wei Xue

**Affiliations:** Department of Urology, Renji Hospital, Shanghai Jiao Tong University School of Medicine, Shanghai, China

**Keywords:** benign prostatic hyperplasia (BPH), single-cell RNA sequencing (scRNA-seq), mendelian randomization (MR), SLC25A37, therapeutic target

## Abstract

**Objectives:**

To identify causal genes for benign prostatic hyperplasia (BPH) based on single-cell RNA sequencing (scRNA-seq) and genome-wide association studies.

**Methods:**

We explored scRNA-seq datasets to identify the differentially expressed genes (DEGs) in BPH patients vs. healthy controls. Mendelian randomization (MR) was conducted to investigate the causal relationships between the identified DEGs and BPH. Bayesian colocalization and reverse MR were performed to consolidate the MR findings. Potential therapeutic drugs targeting causal genes were explored via molecular docking. The results of bioinformatics analyses were validated through experiments including flow cytometry, quantitative reverse transcription polymerase chain reaction, western blotting, immunohistochemistry, and immunofluorescence staining.

**Results:**

BPH patients showed an increased proportion of myeloid cells in the prostate transition zone, with 85 classical monocyte-specific DEGs. Among these, SLC25A37 was causally associated with BPH based on MR, Bayesian colocalization, and reverse MR analyses. Functional analyses indicated its involvement in proliferation-related signaling pathways in classical monocytes. Ferriheme chloride and catechol were identified as potential drugs targeting SLC25A37. *In vitro* study confirmed increased expression levels of SLC25A37 and myeloid cells in BPH tissues.

**Conclusions:**

Our integrative analyses revealed SLC25A37 as a novel causal gene in BPH pathogenesis, unveiling potential therapeutic strategies for BPH treatment.

## Introduction

Benign prostatic hyperplasia (BPH) is a common urological disease among the aging male population with increasing prevalence [1]. According to the Global Burden of Disease data, the incidence cases of BPH increased by 105.70 % from 1990 to 2019 [2], and the lifetime prevalence of BPH was 26.20 % reported by a systematic review containing 31 studies [3], causing the burden of BPH to rise in most of the world [4]. BPH occurs when stromal and epithelial cells in the transitional zone of the prostate proliferate, causing lower urinary tract symptoms (LUTS) including urgency, frequency, nocturia, and incomplete urination, which affect individuals’ quality of life [5]. In addition, recent studies have revealed a correlation between BPH and prostate cancer, chronic kidney disease, and depression [6].

Despite years of research on the pathogenesis of BPH, the etiology remains to be elucidated. Epidemiological data suggest that chronic inflammation is an important contributor to the pathogenesis of BPH, which can be triggered by stimuli including infection, metabolic syndrome, the aging process, and autoimmune response [7]. Immune cells, especially T lymphocytes, are found to accumulate in the prostate inflammatory microenvironment and interact with stromal cells through proinflammatory cytokines and chemokines [1], 8]. Lipid-enriched macrophage was identified as a regulator of increasing prostate size in BPH [9]. However, the detailed roles of each type of immune cell in BPH still need to be deeply elucidated. Single-cell RNA sequencing (scRNA-seq) technology is a powerful tool for analyzing mechanisms at the individual cell level and has been applied to BPH studies [10], 11].

Furthermore, observational studies have indicated the relationship between BPH and common risk factors including age, obesity, diabetes, hypertension, diet, sex hormone levels, and genetics [1]. However, these studies cannot completely eliminate the influence of uncontrolled confounders, reverse causality, and other biases. Mendelian randomization (MR) is a contemporary statistical method to explore the causal relationship between exposure and outcome. MR employs single-nucleotide polymorphisms (SNPs) as genetic instrumental variables from genome-wide association studies (GWAS), which can reduce the influence of bias since genetic variants are randomly allocated at conception and not altered by disease progression [12], [13], [14]. Nonetheless, GWAS studies alone might also be insufficient to identify disease-causing genes, since 90 % of GWAS-identified genetic variants are in noncoding regions of the genome [15], 16]. These genomic regions are enriched in expression quantitative trait loci (eQTLs) [17], which indicates the genomic loci are associated with the expression levels of genes. Since GWAS identifies variants associated with disease risk and eQTLs are related to gene-expression levels, colocalization of GWAS and eQTL can identify whether genetic signals that influence the phenotype and gene expression share the same genetic locus, which could be more powerful in exploring potential causal genes related to diseases [18], 19].

**Figure 1:**
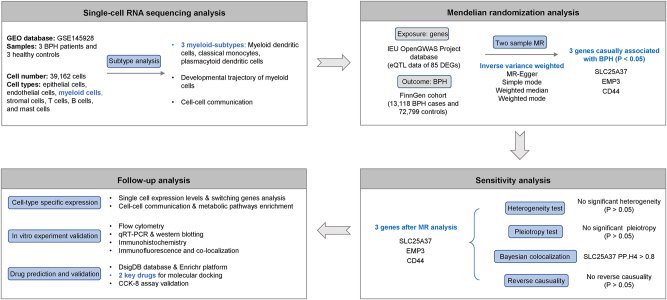
Overview of the study design.

In this study, we performed integrated analyses of scRNA-seq of the prostate transition zone tissues and MR to reveal the causal effect of potential genes on BPH. The overview study design was illustrated in Figure 1. First, we analyzed scRNA-seq data and identified different single-cell clusters and the proportion of each cell type in prostate transition zone tissues of BPH patients and healthy controls. Second, we focused on myeloid cells, which increased significantly in BPH patients, explored their subtypes and subtype-specific DEGs, and derived their eQTL data for causal effect analysis. Third, we performed MR, Bayesian colocalization, and reverse MR analyses to identify potential genes associated with BPH. Finally, we analyzed the expression and function of the candidate genes at the single-cell level, exploring the cell-cell interaction and metabolic pathways to deepen the insights into the effect of these candidate genes on BPH. Further interaction network analyses and *in vitro* experiments were conducted to strengthen our findings.

## Materials and methods

### ScRNA-seq analysis

The scRNA-seq data relating to human BPH patients and healthy controls were derived from the Gene Expression Omnibus (GEO) database under the accession ID GSE145928 (https://www.ncbi.nlm.nih.gov/geo/query/acc.cgi?acc=GSE145928, accessed on Apr 30, 2020) from Joseph et al. [20]. We selected transition zone cells for analysis and carried out data preprocessing and transformation using the “Seurat” R package [21]. We utilized the Harmony algorithm to integrate the datasets, effectively removing technical variations between the samples. For dimensionality reduction, we first performed Principal Component Analysis (PCA), with dims 1:15. We then selected the top 30 principal components (PCs) for downstream analyses. For the cell clustering step (using the FindClusters function in the Seurat package), we used a resolution parameter of 1.0.

We then inferred cell lineages and a pseudo-timeline of myeloid cells through the “slingshot” R package [22] since its flexibility in handling potential multi-lineage differentiation made it particularly suitable for our analysis, and we performed cell-cell communication analysis utilizing the “CellChat” R package [23]. To examine the highly expressed genes in the classical monocytes, we took the intersection of myeloid cell-specific DEGs and the classical monocyte-specific DEGs compared with other cell types. The DEGs were identified based on log_2_ fold change (log_2_FC) >0.5 and adjusted p-value<0.05 after Bonferroni correction.

### Exposure and outcome data

Participants in both the exposure and outcome cohorts were of European ancestry, and no individuals were shared across the two datasets. We used the “org.Hs.e.g.db” R package to convert the gene ID of DEGs to Ensembl ID. Subsequently, the eQTL data of DEGs were collected from the Integrative Epidemiology Unit (IEU) OpenGWAS Project database (https://gwas.mrcieu.ac.uk/). The outcome cohort of BPH (ID: finn-b-N14_PROSTHYPERPLA, with 13,118 BPH cases and 72,799 controls) extracted from the FinnGen cohort was employed as the outcome data, which included information on 16,378,414 SNPs. The data were easily accessible as VCF files for MR analysis.

### MR analysis

Based on the exposure and outcome data, we investigated the potential roles of DEGs in BPH using MR. We began by selecting independent SNPs associated with the eQTLs as genetic instruments. The genetic instruments used in MR must rely on three core assumptions: (1) They must be strongly associated with the exposure. (2) They should be independent of any confounder of the exposure-outcome association. (3) They are assumed to exert no direct effect on the outcome, influencing it only through the exposure. These assumptions imply that the instruments have a causal effect on the outcome only via the risk factor [24]. SNPs were excluded on the basis of either failing to meet the genome-wide significance threshold 5 × 10^−8^ or having a minor allele frequency (MAF) below 0.01. After harmonizing the exposure and outcome summary data, we selected independent SNPs without linkage disequilibrium (LD) (*R* ^2^<0.01 with strand alignment=10,000 kb). The genetic instruments with an *F*-statistic <10 were removed.

Finally, the “TwoSampleMR” R package [25] was employed to evaluate causal inference between the exposure and outcome. For the main analysis, we employed the Wald ratio method for genes with only one SNP, while for genes with two or more instrumental SNPs, estimates were obtained using the inverse variance weighted (IVW), MR Egger, simple mode, weighted mode, and weighted median method. If all genetic variants satisfied the instrumental variable assumptions, then the IVW method was considered the most powerful method to estimate the causal effect and was used as the primary analysis in our study. For multiple testing corrections, the Bonferroni correction was applied consistently across all primary MR analyses, with the significance threshold adjusted based on the number of tested phenotypes or genetic instruments. We also assessed the potential reverse causation of the outcome of the exposure to ensure the accuracy of the results.

### Bayesian colocalization analysis

Considering that MR alone might be insufficient in identifying credible genes on causal pathways to BPH, Bayesian colocalization analysis based on the “coloc” R package [19] was performed to assess whether the expression of eQTLs and BPH genetic associations shared genetic signals in a genomic region. Genomic regions were defined as 100 kb upstream and downstream of the transcription start site of each locus. As described previously [26], Bayesian colocalization analysis included five hypotheses (H0–H4) on whether a single variant was shared between two traits. In this study, we tested the posterior probability of H4 (PP.H4), which represented that there was a shared genetic signal for the expression of eQTLs and BPH genetic associations. The PP.H4>80 % was considered to indicate strong evidence of colocalization.

### Interaction network analyses of candidate genes

To further validate the association between our candidate genes and the pathogenesis of BPH, we used GeneMANIA (http://www.genemania.org) to construct the interaction between candidate genes and other pathogenic genes of BPH. STRING database (http://string-db.org) [27] and Cytoscape software (version 3.10.1) were utilized for constructing protein-protein interaction (PPI) networks between BPH therapy targets and candidate genes-interacting proteins.

### Identification of candidate drugs and molecular docking

The Drug Signatures Database (DSigDB) and Enrichr platform (https://maayanlab.cloud/Enrichr) were used to identify potential drugs targeting candidate genes and their interacting genes, which were accessed through the GeneMANIA website. The top 10 compounds with the lowest adjusted p-values were selected as candidate drugs. We further performed molecular docking at the atomic level based on AutodockVina 1.2.2 (http://autodock.scripps.edu/), a computerized protein-ligand docking software. The molecular structure of the candidate drug was downloaded from the PubChem Compound database (https://pubchem.ncbi.nlm.nih.gov/) and converted to PDBQT format. The protein structure data of the target protein were retrieved from the Protein Data Bank database (http://www.rcsb.org/). Initially, all water molecules were removed from the protein and ligand data files, and polar hydrogen atoms were added. The grid boxes were centered to cover the structural domains of each protein and to facilitate the movement of the free molecule. A grid size of 30 Å × 30 Å × 30 Å was used to build up the docking pocket, and the grid point distance was set to 0.05 nm. The methods of the following *in vitro* experiments were shown in Supplementary Methods.

### Statistical analysis

We employed R software (version 4.2) and SPSS^®^ version 26.0 (IBM, Armonk, New York, USA) to perform all statistical analyses. The *F*-statistic should be greater than 10 to avoid the weak instrumental variable bias. p-value <0.05 was considered for the causal relationships between the two traits. The expression levels of SLC25A37 in myeloid cells in the prostate transition zone between BPH patients and healthy controls were evaluated by the Wilcoxon test.

**Ethical approval:** The acquisition of human prostate tissue in this study was approved by the Ethics Committee of Renji Hospital, Shanghai Jiao Tong University School of Medicine (Ethical approval number: LY2023-234-C) and was in accordance with the Declaration of Helsinki (as revised in 2013).

## Results

### ScRNA-seq analysis of prostate transition zone tissues in BPH patients and healthy controls

**Figure 2:**
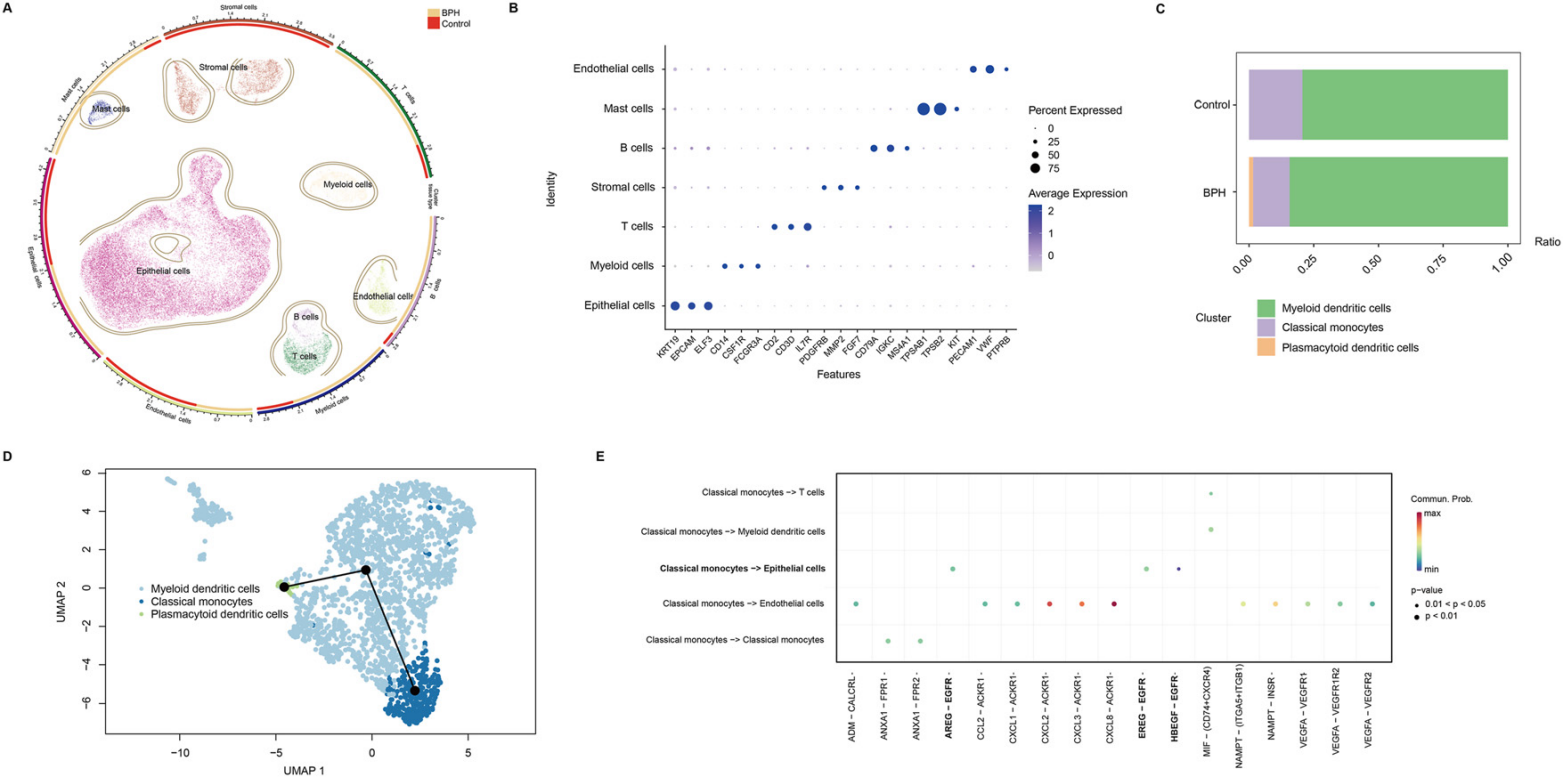
Identification of myeloid subtypes, developmental trajectory, and cell communication. (A) 39,162 cells were classified into 7 types and displayed in UMAP using the “plot1cell” R package. The outer circle showed epithelial cells, endothelial cells, mast cells, B cells, T cells, stromal cells, and myeloid cells. The inner circle showed the relative number of each cell type between BPH patients and healthy controls. (B) Dot plot of cluster-specific genes of the 7 cell types. (C) The stack bar plot demonstrated the proportion of 3 subtypes of myeloid cells in BPH patients and healthy controls, with color coding according to each cluster. (D) We identified the developmental trajectory of myeloid cells using the “slingshot” package. (E) The bubble plot showed all the significant ligand-receptor pairs contributing to the signaling sending from classical monocytes to other cells, the dot size representing the p-values, colored by the calculated communication probabilities.

Three BPH patients and three healthy controls in the GSE145928 database were selected for scRNA-seq analysis. After standard quality control mentioned in the “Materials and Methods” section, a total of 39,162 single cells were included for downstream analyses. Cells were clustered into 7 types (epithelial cells, endothelial cells, myeloid cells, stromal cells, T cells, B cells, and mast cells) (Figure 2A) according to cluster-specific genes (Figure 2B). The number and proportion of each cell type were shown in Supplementary Table S1. We observed that myeloid cells and T cells were the most abundant immune populations in BPH (Figure 2A). Given that the proportion of myeloid cells (marked by CD14, CSF1R, and FCGR3A) displayed a more prominent increase in the BPH group compared with the healthy control group, we chose this population for in-depth analysis. Further analyses demonstrated that the myeloid cells were clustered into 3 subtypes after annotation (classical monocytes, myeloid dendritic cells, and plasmacytoid dendritic cells). The proportion of classical monocytes decreased in BPH patients, while the proportions of myeloid dendritic cells and plasmacytoid dendritic cells increased (Figure 2C).

Subsequently, we identified the developmental trajectory of myeloid cells, indicating that the classical monocytes might be the less differentiated, early-state population, which developed into myeloid dendritic cells, and then plasmacytoid dendritic cells according to the lineage inference (Figure 2D). We then focused on this subtype and revealed its communication with other cell types to shape the microenvironment in BPH. Using the “CellChat” R package, we found the cell-cell communication between classical monocytes and other cells including T cells, epithelial cells, endothelial cells, and myeloid dendritic cells (Figure 2E). Notably, the classical monocytes interacted with epithelial cells through EGFR (Epidermal Growth Factor Receptor)-related pathways. (Figure 2E).

### MR analysis identified the causal effects of 3 potential genes on the pathogenesis of BPH

To investigate the relationship between classical monocytes and the pathogenesis of BPH, we took the intersection of myeloid cell-specific and classical monocyte-specific DEGs, identified 85 genes, as shown in Supplementary Table S2. We obtained eQTL data of these genes and identified 367 SNPs of eQTLs and 268 SNPs of BPH from the IEU OpenGWAS database after selection in the “Materials and Methods” section. The exposure and outcome data were summarized in Supplementary Tables S3–S4.

**Figure 3:**
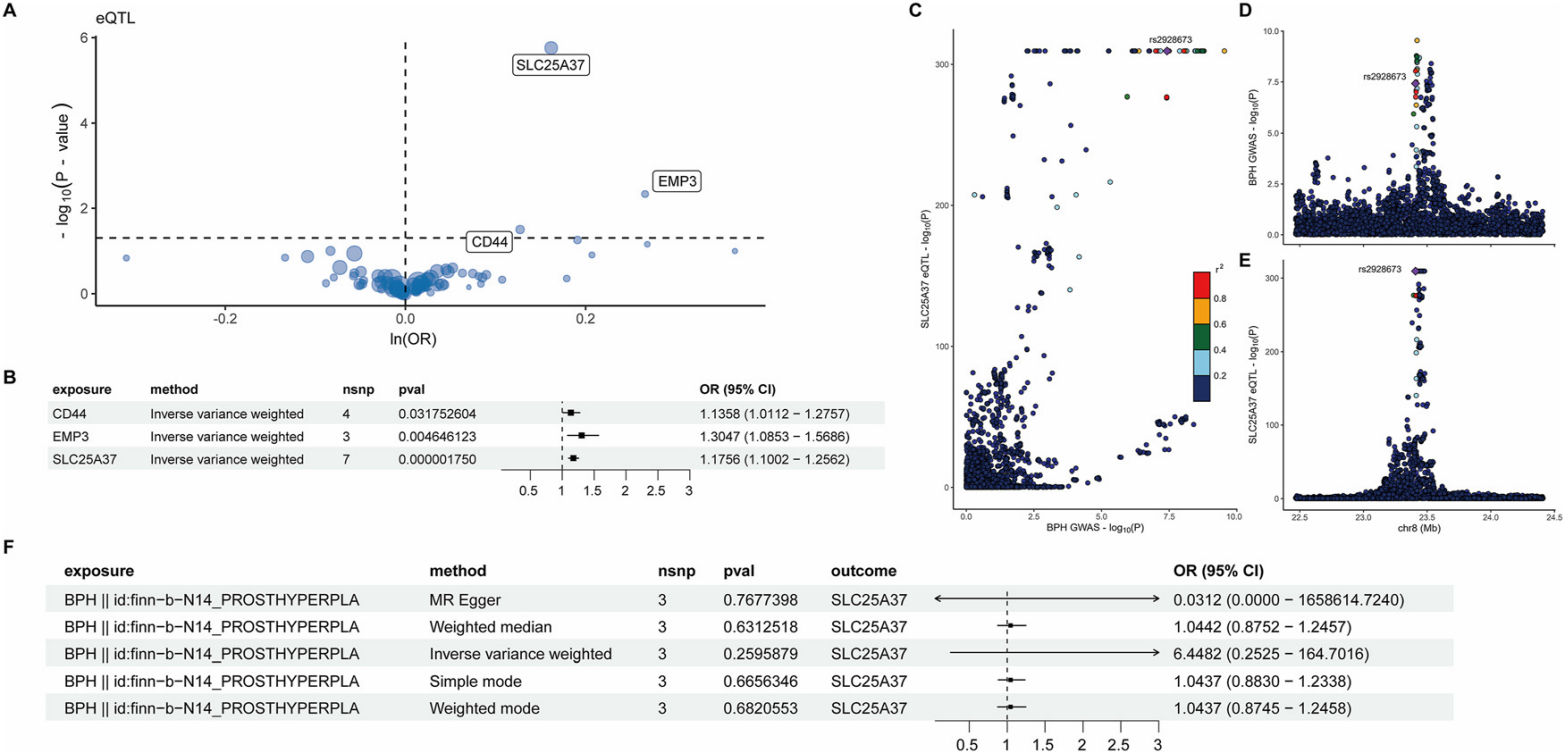
MR, reverse MR, and colocalization results. (A) The volcano plot showed results from mendelian randomization, the dot size representing PVE. (B) The forest plot indicated the causal effects of CD44, EMP3, and SLC25A37 on the incidence of BPH. (C)–(E) Bayesian colocalization analysis between eQTLs and BPH genetic association data. (C) Locus comparison showed a colocalization signal for SLC25A37, with rs2928673 having the lowest p-value for both eQTL and BPH genetic association among the 3 genes. (D) Genetic association signal for the GWAS data of BPH. (E) Genetic association signal for the eQTL of SLC25A37. (F) The forest plot evaluated the reverse causation of BPH on SLC25A37 eQTL. OR: odds ratio; 95 % CI: 95 % confidence interval.

To gain insights into the causal effects of DEGs on BPH, we performed a two-sample MR analysis between the 85 genes and BPH. The results were summarized in Supplementary Table S5, indicating that elevated levels of CD44, EMP3, and SLC25A37 were causally associated with a higher incidence of BPH (Figure 3A and Table 1), a forest plot summarizing the results was shown in Figure 3B. To address multiple comparisons, we applied the Bonferroni correction to adjust our results, setting the significance threshold set at 0.05/(the total number of proteins analyzed). Odds ratios (OR) for the genes above were 1.14 (95 % confidence interval (CI), 1.01–1.28), 1.30 (95 % CI, 1.08–1.57), and 1.18 (95 % CI, 1.10–1.26), respectively. The *F*-statistic for each individual SNP used as an instrument was calculated. The minimum *F*-statistic across all instruments was 31.25, substantially above the conventional threshold of 10, indicating that potential weak instrument bias is minimal and unlikely to have a significant impact on the causal estimates reported in our MR analysis. No significant heterogeneity or pleiotropy was found among the three genes (*P*_heterogeneity_>0.05, *P*_pleiotropy_>0.05) (Supplementary Tables S6–S7).

**Table 1:** MR results for genes significantly associated with the incidence of BPH.

	Gene	Ensembl ID	SNP	Effect allele	OR (95 % CI)	p-Value	PVE*	*F-*statistic
1	CD44	ENSG00000026508	rs1375493	A	1.14 (1.01, 1.28)	3.18E−02	1.63 %	69.38
2	CD44	ENSG00000026508	rs507230	A	1.14 (1.01, 1,28)	3.18E−02	1.63 %	145.37
3	CD44	ENSG00000026508	rs286873	T	1.14 (1.01, 1.28)	3.18E−02	1.63 %	35.49
4	CD44	ENSG00000026508	rs927335	T	1.14 (1.01, 1.28)	3.18E−02	1.63 %	266.59
5	EMP3	ENSG00000142227	rs597808	G	1.30 (1.09, 1.57)	4.65E−03	0.69 %	53.05
6	EMP3	ENSG00000142227	rs35970658	G	1.30 (1.09, 1.57)	4.65E−03	0.69 %	31.25
7	EMP3	ENSG00000142227	rs8102550	C	1.30 (1.09, 1.57)	4.65E−03	0.69 %	122.23
8	SLC25A37	ENSG00000147454	rs1354034	C	1.18 (1.10, 1.26)	1.75E−06	6.63 %	50.86
9	SLC25A37	ENSG00000147454	rs590856	A	1.18 (1.10, 1.26)	1.75E−06	6.63 %	44.04
10	SLC25A37	ENSG00000147454	rs7819409	T	1.18 (1.10, 1.26)	1.75E−06	6.63 %	378.72
11	SLC25A37	ENSG00000147454	rs6557629	G	1.18 (1.10, 1.26)	1.75E−06	6.63 %	40.35
12	SLC25A37	ENSG00000147454	rs2928673	T	1.18 (1.10, 1.26)	1.75E−06	6.63 %	1,451.68
13	SLC25A37	ENSG00000147454	rs1196092	G	1.18 (1.10, 1.26)	1.75E−06	6.63 %	72.70
14	SLC25A37	ENSG00000147454	rs225245	G	1.18 (1.10, 1.26)	1.75E−06	6.63 %	63.54

*PVE, proportion of variance explained.

### Colocalization analyses supported the causality of SLC25A37 with BPH

As an additional measure of sensitivity, we performed Bayesian colocalization analyses between the eQTLs for CD44, EMP3, and SLC25A37 and BPH association data. Of the three genes identified by MR, SLC25A37 was supported by strong evidence of genetic colocalization (Table 2), indicating high posterior probability (PP.H4=89.9 %) for a shared genetic signal between eQTL and BPH. For SLC25A37, the colocalization signal showed that rs2928673, located at chr8:23551407 (GRCh38.p14), was the shared SNP with the lowest p-value for both the eQTL and BPH genetic association data, while CD44 and EPM3 failed to meet hypothesis 4 (Figure 3C–E). The results indicated that the shared SNP could regulate SLC25A37 expression as well as drive the association with BPH. To assess potential reverse causation, whereby BPH influenced SLC25A37, we performed bidirectional MR that used BPH as the exposure and SLC25A37 as the outcome. No reverse causation was found using five methods (Figure 3F). Therefore, we found SLC25A37 as the most likely causal gene, and our subsequent analyses focused on SLC25A37 at the single-cell level.

**Table 2:** The colocalization results of eQTLs and BPH genetic association data.

Trait 1	Trait 2	PP.H0	PP.H1	PP.H2	PP.H3	PP.H4
CD44-eQTL	CD44-GWAS	1.90e−53	7.93e−54	0.700	0.292	0.008
EPM3-eQTL	EPM3-GWAS	3.75e−22	8.00e−23	0.767	0.164	0.070
SLC25A37-eQTL	SLC25A37-GWAS	2.53e−309	2.68e−305	9.63e−06	0.101	0.899

### Cell type-specific expression in myeloid cells and interaction network analysis of SLC25A37

**Figure 4:**
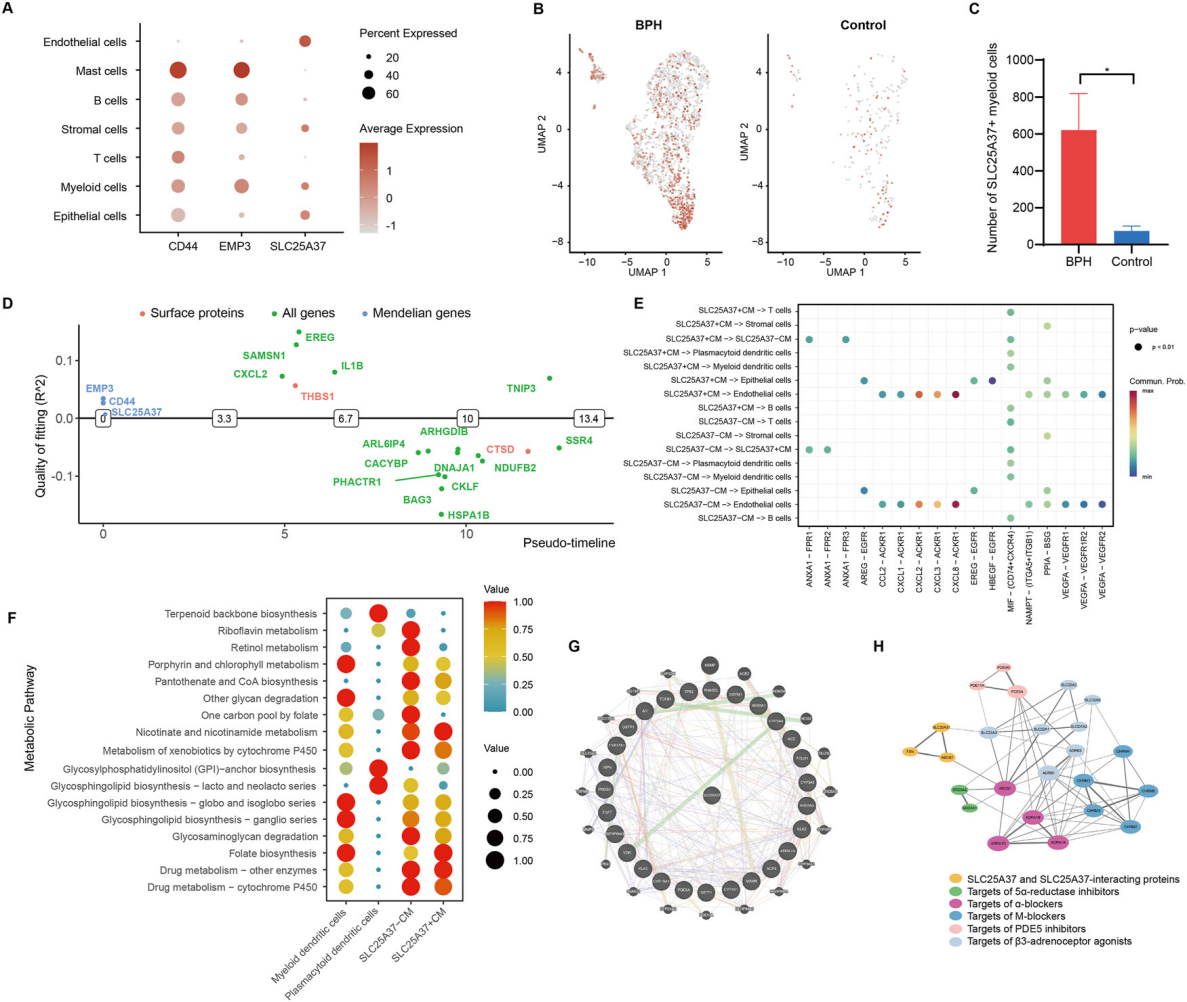
Cell-type specificity expression analysis of candidate genes. (A) Single-cell expression of the 3 genes identified by MR across 7 cell types in BPH patients, the dot size representing the percent of genes enriched in each item, colored by average expression. (B) UMAP plots showed the expression levels of SLC25A37 in myeloid cells, colored by SLC25A37^+^ cells. (C) Bar plots showed the difference in the number of myeloid cells expressing SLC25A37 between BPH patients and healthy controls, ∗ p<0.05, by a two-sided wilcoxon test. (D) Visualization of the order of the top switching genes, our candidate genes, and surface proteins along the cell development pseudo-timeline. (E) The dot plot showed all the significant ligand-receptor pairs contributing to the signaling sending from SLC25A37^+^ classical monocytes and SLC25A37^-^ classical monocytes to different cell types, the dot size representing the p-values, colored by the calculated communication probabilities. CM: classical monocytes. (F) The dot plot showed the metabolic activity analysis of different myeloid subtypes, with both the dot size and color indicating the scaled metabolic score. (G) GeneMANIA database analysis showed that SLC25A37 interacted with BPH pathogenic genes. (H) The PPI network showed the association between SLC25A37 and SLC25A37-interacting proteins and molecular targets of several common drug therapies including 5ɑ-reductase inhibitors, ɑ-blockers, M-blockers, PDE5 inhibitors, and β3-adrenoceptor agonists. SLC25A37-interacting proteins were explored through the GeneCards database. The line thickness indicated the strength of interaction between any two proteins.

To explore whether the candidate genes had any cell type-specific enrichment in BPH, we further analyzed the single-cell expression levels of CD44, EMP3, and SLC25A37 based on the UMAP plot derived from Figure 2A. SLC25A37 was mainly enriched in myeloid cells, endothelial cells, and epithelial cells, while CD44 and EMP3 were mainly enriched in myeloid cells, mast cells, and B cells (Figure 4A). The UMAP plots in Figure 4B showed higher expression of SLC25A37 in myeloid cells in the transition zone in BPH patients compared to healthy controls. Figure 4C demonstrated the comparison of the total number of SLC25A37^+^ myeloid cells between BPH patients and healthy controls. We then investigated the function of SLC25A37 in cell development pseudo-timeline compared with other important switching genes and surface proteins. The result demonstrated that SLC25A37 was switched on in the early stage of the pseudo-timeline, indicating that SLC25A37 played an important part in the early development of classical monocytes (Figure 4D).

Subsequently, we analyzed the signaling pathways from SLC25A37^+^ and classical monocytes to different cell subpopulations, which showed the HBEGF (heparin-binding epidermal growth factor-like growth factor)-EGFR pathway had higher communication probability between SLC25A37^+^ classical monocytes and epithelial cells compared with SLC25A37^-^ classical monocytes (Figure 4E). Furthermore, we explored single-cell metabolic pathway enrichment in different myeloid subtypes (Figure 4F). Notably, we found that SLC25A37^+^ classical monocytes in BPH were strongly enriched in folate biosynthesis and glycosylphosphatidylinositol (GPI) -anchor biosynthesis-related pathways compared with SLC25A37^-^ classical monocytes, which might contribute to cell proliferation in prostate transition zone tissues and lead to BPH.

To investigate the potential association between SLC25A37 and pathogenic genes of BPH, we searched the Phenopedia database and Malacards database and retrieved 27 BPH-related genes. The interaction of SLC25A37 with other genes visualized by GeneMANIA was shown in Figure 4G. We then explored the DrugBank database to identify the molecular targets of several common drug therapies used to treat BPH, and constructed the PPI network between these targets and SLC25A37-related proteins using the STRING database (Figure 4H), indicating these drug therapies might treat BPH by potentially regulating the expression of SLC25A37.

### Comparison of CD14^+^ myeloid cells and SLC25A37 in prostate tissues between the BPH patients and healthy controls

**Figure 5:**
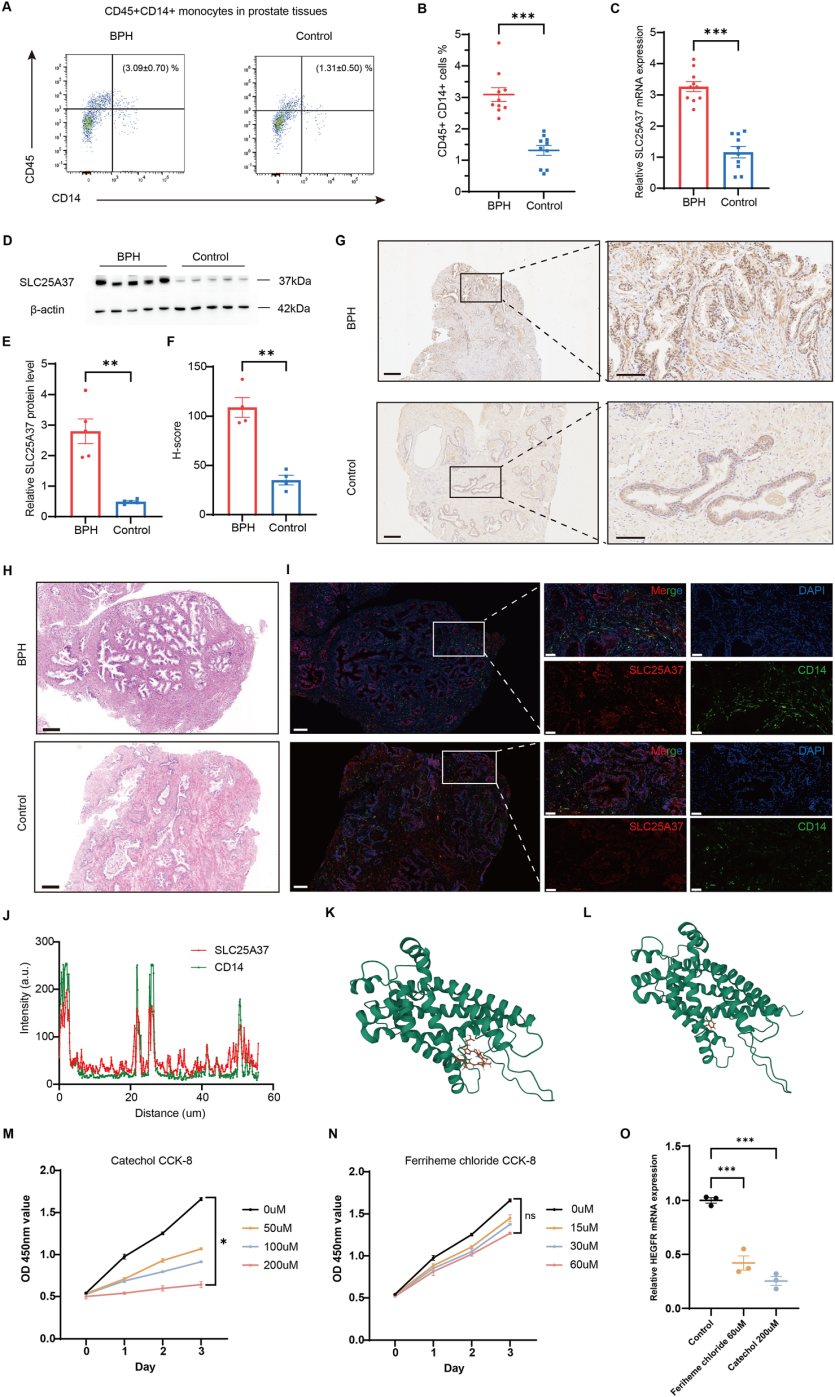
Comparison of CD14^+^ myeloid cells and SLC25A37 between the BPH patients and healthy controls, and validation of candidate drugs through *in vitro* experiments. (A) Flow cytometric analysis of the proportion of CD45^+^CD14^+^ myeloid cells in prostate tissues in BPH patients and healthy controls. (n=10) (B) Comparison of the proportion of CD45^+^CD14^+^ myeloid cells in prostate tissues in BPH patients and healthy controls. (n=10) (C) The relative SLC25A37 mRNA expression between BPH patients and healthy controls evaluated by RT-qPCR. (n=10) (D)–(E) The protein level of expression SLC25A37 between BPH patients and healthy controls was measured by western blotting. (n=5) (F) Comparison of SLC25A37 protein expression in BPH patients and healthy controls by immunohistochemistry. (n=4) (G) Representative IHC images of prostate tissues of BPH patients and healthy controls. Scale bars, 250um (left), and 100um (right), respectively. (H) Representative HE images of prostate tissues of BPH patients and healthy controls. Scale bar, 250um. (I) Representative fluorescence images of BPH patients and healthy controls. Scale bars, 200um (left), and 50um (right), respectively. (J) Colocalization analysis of SLC25A37 and CD14 in prostate tissues. (K) Binding mode of ferriheme chloride to SLC25A37 by AutoDock software. (L) Binding mode of catechol to SLC25A37 by AutoDock software. (M) CCK-8 proliferation assay to detect the impact of catechol on cell proliferation. (N) CCK-8 proliferation assay to detect the impact of ferriheme chloride on cell proliferation. (O) The impact of 60uM ferriheme and 200uM catechol on HEGFR mRNA expression evaluated by RT-qPCR.

To further validate our primary findings through bioinformatics analyses, we collected prostate tissues from BPH patients and healthy controls for *in vitro* experiments, the sample information was listed in Supplementary Table S8. Flow cytometric analysis showed the proportion of CD45^+^CD14^+^ myeloid cells in prostate tissues in BPH patients was significantly higher than that in healthy controls (Figure 5A and B). RT-qPCR and western blotting revealed that the expression of SLC25A37 was higher in the prostate tissues of BPH patients (Figure 5C–E). Immunohistochemistry showed SLC25A37 protein expression in prostate tissues in BPH patients was significantly higher than that in healthy controls (Figure 5F and G). Immunofluorescence images also demonstrated higher expression of CD14 and SLC25A37 in BPH patients (Figure 5H and I), with SLC25A37 and CD14 colocalization in prostate tissues (Pearson correlation coefficient=0.44, Figure 5J).

### Candidate drug prediction and validation

Finally, we explored the DsigDB database and the Enrichr platform to predict potential drugs that could target SLC25A37 and related genes. The top 10 candidate drugs with the lowest adjusted p-values and their detailed information were presented in Table 3, providing potential therapeutic options for BPH. We chose ferriheme chloride (PubChem CID 15953900) and catechol (PubChem CID 289) with the lowest adjusted p-value and relatively higher odds ratio and combined score to perform molecular docking analysis. Each interaction between the candidate drug and SLC25A37 was generated through Autodock Vina v.1.2.2 (Figure 5K and L). Ferriheme chloride and catechol had binding energy of −8.665 and −4.645 kcal/mol, respectively.

**Table 3:** The top 10 candidate drugs targeting SLC25A37.

No.	Drugs	Adjusted p-Value	Odds ratio	Combined score
1	Ferriheme chloride CTD* 00006079	0.006	77.271	859.796
2	Catechol CTD 00001636	0.006	77.271	859.796
3	Nitroglycerin BOSS*	0.006	29.517	316.169
4	Hydrogen sulfide BOSS	0.006	26.324	270.519
5	Iron BOSS	0.006	25.737	262.288
6	Betamethasone CTD 00005504	0.019	140.098	1,241.768
7	Phenol CTD 00007305	0.019	33.132	288.580
8	Ademetionine CTD 00006715	0.037	84.017	665.560
9	Cobalt sulfate CTD 00001238	0.046	69.996	530.512
10	Adenosine diphosphate BOSS	0.050	63.624	470.753

*BOSS, biomedical object search system; CTD, comparative toxicogenomics database.

In addition, we performed a CCK-8 proliferation assay to detect the impact of ferriheme chloride and catechol on BPH-1 cell proliferation (Figure 5M and N). We found that targeting SLC25A37 by catechol could significantly inhibit the proliferation of BPH-1 cells. To validate the mechanism in Figure 4E, we performed RT-qPCR experiments and found that inhibiting SLC25A37 significantly by 60uM ferriheme or 200uM catechol decreased the mRNA expression of HBEGF (Figure 5O). These results suggested that SLC25A37 contributed to BPH pathogenesis by upregulating HBEGF, which activated the EGFR signaling pathway to drive the epithelial cell proliferation characteristic of BPH.

## Discussion

This study integrated analyses of scRNA-seq and MR, providing evidence that the SLC25A37 gene was causally associated with the pathogenesis of BPH. To the best of our knowledge, our study first demonstrated the causal effect of SLC25A37 on BPH. SLC25A37, also known as mitoferrin-1 (Mfrn 1), is a member of solute carrier (SLC) transporters. SLC25A37 encodes a member of small molecular carriers located in the mitochondrial inner membrane, functioning as an iron importer for the synthesis of mitochondrial heme and iron-sulfur cluster [28]. Recent studies found the dysregulation of SLC25A37 was associated with clear-cell renal cell carcinoma [29], pancreatic cancer [30], and osteosarcoma [31]. The function of SLC25A37 could explain our findings since the upregulated expression level of SLC25A37 might promote cell proliferation of stromal and epithelial cells in the prostate and lead to higher BPH incidence.

In our study, we mainly focused on the expression level and function of SLC25A37 in myeloid cells since the monocyte-macrophage system is one of the typical infiltrating immune cells in BPH specimens [32]. We found signaling pathways from SLC25A37^+^ classical monocytes to epithelial cells were highly enriched in the HBEGF-EGFR pathway compared with SLC25A37^-^ classical monocytes. HBEGF had been demonstrated as an endogenous stromal growth factor that acted as a mediator of angiotensin II to regulate epithelial cell proliferation and BPH development [33]. Besides, we employed metabolic pathway analysis to deepen the insights into the functions of SLC25A37^+^ classical monocytes. Notably, the folate biosynthesis and GPI-anchor biosynthesis-related pathways were strongly enriched compared with SLC25A37^-^ classical monocytes. Folate plays an essential role in the one-carbon metabolism process, which is responsible for nucleotide synthesis and biological methylation reactions [34]. Hence, the alteration of folate metabolism has been widely investigated as a possible mechanism for cancer development [35]. Jeffrey et al. found higher serum folate concentration was associated with increased prostate cancer cell proliferation [36], which might reflect that excessive folate provides abundant DNA synthesis for the rapid proliferation of cancer cells. Renee et al. also indicated that folic acid supplementation and higher serum levels were associated with an increased risk of prostate cancer [37]. However, folate might prevent tumor development by enhancing genomic DNA stability, revealing its dual role in carcinogenesis [38]. Few studies have investigated the relationship between folate and BPH, and our analysis suggested that the SLC25A37^+^ classical monocytes had higher activity to synthesize folate, which might be attributed to their higher requirement for cell proliferation. Besides, GPI-anchor is a protein on the cell membrane that performs biological functions including lipid raft partitioning, signal transduction, and targeting the apical membrane [39]. At least 150 human proteins are GPI-anchored proteins [40], and some tumor biomarkers including carcinoembryonic antigen (CEA) are attached by GPI anchors [41]. The dysregulation of these GPI anchors might be associated with abnormal cell proliferation and tumorigenesis [42]. The function of GPI-anchor biosynthesis in SLC25A37^+^ classical monocytes might be related to the activity of epithelial cell proliferation in the prostate as well. Finally, we predicted 10 potential drugs targeting SLC25A37 and its related genes, which might provide new possibilities for the treatment of BPH and need further investigation into the mechanisms of these novel therapeutic options.

The present work had several limitations. First, our sample size of 3 BPH patients and 3 healthy controls is relatively small. While scRNA-seq provides deep transcriptional profiling at the single-cell level within each sample, the limited cohort size may not fully capture the broad biological heterogeneity within the BPH patient population mcaXZ*nd healthy controls. Since our findings provide insights into the mechanism of BPH, a larger cohort would be necessary to validate the identified cell populations and molecular signatures across a broader patient population. Second, our GWAS and MR analyses were restricted to individuals of European ancestry, which may limit the direct generalizability of the genetic associations. Though the core findings were subsequently experimentally validated in models and patient samples of Asian ancestry, future genetic studies in multi-ethnic cohorts are warranted to fully confirm the transferability of the specific genetic instruments and effect sizes. Third, our study focused on the function of myeloid cells in BPH pathogenesis, the specific roles of other infiltrating immune cells, such as T cells, need deeper investigation in future work. Fourth, we found SLC25A37 contributed to BPH pathogenesis by upregulating HBEGF/EGFR signaling pathway to drive the epithelial cell proliferation, but the specific cell-to-cell interaction between the SLC25A37^+^ monocyte and the epithelial cell requires further complex co-culture studies. Given that SLC25A37 was also found to be enriched in endothelial and stromal cells, our current results could not evaluate the specific functional role of SLC25A37 within these other cell populations or their distinct contributions to the pathogenesis of BPH. Future studies are also necessary to confirm direct physical binding between potential drugs and SLC25A37.

## Conclusions

In summary, this study revealed the causal effect of the SLC25A37 gene on the pathogenesis of BPH based on MR integrating eQTL and GWAS data. The scRNA-seq analysis indicated that in the prostate transition zone of BPH patients, SLC25A37 was upregulated in myeloid cells, especially classical monocytes, and involved in cell proliferation-related signaling pathways, providing novel insights into the underlying mechanism of BPH and possible novel therapeutic options.

## Supplementary Material

Supplementary Material

Supplementary Material

Supplementary Material
