# Supplementary material for: SLC25A37 as a novel therapeutic target for benign prostatic hyperplasia: integrative analyses of single-cell RNA sequencing and genome-wide association studies
Source: Open Med (Wars). 2026 Jan 13;21(1):20251371. doi: 10.1515/med-2025-1371 (PMC12917586; doi:10.1515/med-2025-1371)
Supplement: Supplementary file 1 — Supplementary Material [file j_med-2025-1371_suppl_001.docx]

**Supplementary Material**

**Supplementary tables: Table S1.** The number and proportion of 7 cell types in the BPH group and the control group according to scRNA-seq cell annotation. **Table S2.** The intersection of monocyte-specific and classical monocyte-specific DEGs. **Table S3.** The exposure data after selection. **Table S4.** The outcome data after selection. **Table S5.** MR results. **Table S6.** Heterogeneity analyses of Mendelian randomization. **Table S7.** Pleiotropy analyses of Mendelian randomization. **Table S8.** Clinical characteristics of BPH patients and healthy controls for prostate sample collection and subsequent in vitro experiments.
