# Supplementary material for: SLC25A37 as a novel therapeutic target for benign prostatic hyperplasia: integrative analyses of single-cell RNA sequencing and genome-wide association studies
Source: Open Med (Wars). 2026 Jan 13;21(1):20251371. doi: 10.1515/med-2025-1371 (PMC12917586; doi:10.1515/med-2025-1371)
Supplement: Supplementary file 2 — Supplementary Material [file j_med-2025-1371_suppl_002.docx]

**Supplementary Methods**

**1 Patients and sample collection**

The acquisition of human prostate tissue was approved by the Ethics Committee of Renji Hospital after obtaining informed consent from the patients. BPH tissues were obtained from patients receiving holmium laser enucleation of the prostate (HoLEP), whose preoperative prostate volume was greater than 50ml based on magnetic resonance imaging (MRI).

Tissues of healthy controls were obtained from the transition zone adjacent to cancer obtained from prostate cancer patients undergoing radical prostatectomy, with preoperative prostate volume less than 30ml based on MR, prostate-specific antigen (PSA) level < 10ng/ml, Gleason score < 8, and clinical T stage < 3. The calculation method of prostate volume is prostate length*width*height*0.52. The age of patients in both groups was between 60 and 80 years old, and there was no significant difference between the BPH and healthy groups. The collected specimens were subjected to H&E (Hematoxylin and Eosin) staining, and pathology did not reveal the presence of prostate cancer according to an experienced pathologist. Tissues for flow cytometry were minced, then digested at 37°C for 30min in RPMI 1640 (Thermo Fisher Scientific) with 20 mg/mL Collagenase D (Roche) and 40 U/mL DNase I (Roche). Digested samples were passed through a 70 μm cell strainer and washed with PBS.

**2 Flow cytometry**

The prostate single cell suspension was centrifuged, the supernatant was removed, and 1mL PBS was added to resuspend. Add 1 μL Fixable Viability Stain 780 (BD Biosciences) to the cell suspension and incubate at room temperature away from light for 15 minutes to discriminate between live and dead cells. Centrifuge the suspension and remove the supernatant, then add 1ml stain buffer (Absin) to resuspend. Add 2 μL CD45-APC, CD14-FITC flow antibodies (Absin) to the cell suspension and incubate at room temperature away from light for 10 minutes. After incubation, centrifuge to remove the supernatant, add 100 μL stain buffer to resuspend, and use a Beckman Coulter flow cytometer to detect the proportion of myeloid cells. The results were analyzed using Flowjo V10.8.1 flow cytometry analysis software.

**3 qRT-PCR analysis**

Total RNA was isolated from cells using TRIzol Reagent (99940001, Invitrogen), and the RNA concentrations were measured using a NANODROP 2000c spectrophotometer (Thermo Fisher Scientific). Reversed transcription was conducted utilizing the Reverse Transcription Kit (R323-01, Vazyme, China). Quantitative PCR (qPCR) was executed with SYBR-green (Q711-02, Vazyme, China) on a LightCycler 480 qPCR machine (Roche). The relative mRNA expression levels were assessed using the 2−ΔΔCq approach. The primer sequences used to amplify the target genes were listed as follows: human SLC25A37 (forward primer sequence: GATGGGGACAGCCGAGATG, reverse primer sequence: ACCGGGTACATGACCGAGT), human HBEGF (forward primer sequence: TGTATCCACGGACCAGCTGCTA, reverse primer sequence: TGCTCCTCCTTGTTTGGTGTGG), human GAPDH (forward primer sequence: CCACCCATGGCAAATTCC, reverse primer sequence: GATGGGATTTCCATTGATGACA).

**4 Protein extraction and western blotting analysis**

Total proteins were extracted using RIPA lysis buffer (Thermo Fisher Scientific) and quantified by BCA Protein Assay Kit (Thermo Fisher Scientific). Equal amounts of total proteins were separated by SDS-PAGE and transferred to polyvinylidene difluoride (PVDF) membranes, which were then blocked with 5% skimmed milk powder to block the nonspecific bindings. Subsequently, the PVDF membranes were incubated with corresponding primary antibodies at 4 °C overnight and then incubated with secondary antibodies conjugated to horseradish peroxidase at room temperature for 1 h. The primary antibodies used in this study included: anti-human SLC25A37 (1:1000, Abmart), anti-human β-actin (1:1000, Cell Signaling Technology). The secondary antibody was anti-rabbit IgG, HRP-linked Antibody (1:2000, Cell Signaling Technology). Enhanced chemiluminescence detection (Millipore, Burlington, MA, USA) was used to measure the indicated protein bands. The quantification of the images was conducted by ImageJ (NIH, Bethesda, MD, USA).

**5 Immunohistochemistry**

The immunohistochemical procedure was performed as follows: formalin-fixed, paraffin-embedded prostate tissue sections were rehydrated through a graded series of xylene and ethanol, followed by antigen retrieval under appropriate conditions. After cooling and washing with PBS, endogenous peroxidase activity was blocked by incubation with 3% hydrogen peroxide. Sections were then blocked with 3% BSA and incubated overnight at 4°C with the SLC25A37 primary antibody (1:400, Abmart). Following PBS washes, the sections were incubated with an HRP-conjugated secondary antibody corresponding to the primary antibody host species. After additional washes, immunoreactivity was visualized using DAB substrate under microscopic control, and the reaction was stopped by rinsing with water. Counterstaining was performed with hematoxylin, followed by differentiation and bluing steps. Finally, sections were dehydrated through an ethanol series, cleared in xylene, and mounted with a coverslip for microscopic examination. The data analysis was conducted using ImageJ software (version 1.8.0).

**6 Immunofluorescence**

Sections of formalin-fixed, paraffin-embedded prostate tissues were dewaxed in xylene, rehydrated through an alcohol gradient, and subjected to antigen retrieval in EDTA buffer (pH 8.0). Subsequently, sections were incubated with primary antibodies against SLC25A37 (1:200, Abmart) and CD14 (1:500, Proteintech) at 4°C overnight, then with corresponding secondary antibody for 1 h at room temperature. DAPI (WAS13011, Wasci) was used to stain the nuclei in the sections. The IF staining images were reviewed using SlideViewer (version 2.5.0.143918), and the co-localization analysis was conducted using ImageJ software (version 1.8.0).

**7 CCK-8 proliferation assay**

The human benign prostatic hyperplasia epithelial cell line BPH-1 (obtained from the cell bank of the Shanghai Biology Institute, Shanghai, China) was cultured in RPMI1640 medium supplemented with 10% fetal bovine serum and 1% penicillin-streptomycin, and maintained at 37 °C in a humidified incubator with 5% CO₂. For cell proliferation analysis, a Cell Counting Kit-8 (CCK-8) (obtained from Absin company, Shanghai, China) assay was employed. In brief, BPH-1 cells were seeded into 96-well plates and treated with different concentrations of catechol or ferriheme chloride, growing for 0, 24, 48, and 72 hours. At each time point, CCK-8 solution was added to each well at a 1:10 dilution and incubated for 2 hours. The absorbance of each well was then measured at 450 nm using a microplate reader, with three replicate wells used per condition for each time point.
